# The dimensionality of niche space allows bounded and unbounded processes to jointly influence diversification

**DOI:** 10.1038/s41467-018-06732-x

**Published:** 2018-10-15

**Authors:** Matthew J. Larcombe, Gregory J. Jordan, David Bryant, Steven I. Higgins

**Affiliations:** 1 Department of Botany, University of Otago, PO Box 56,, Dunedin, 9054 New Zealand; 2 Biological Sciences, University of Tasmania, Private Bag 55, Hobart, Tasmania 7001 Australia; 3 Department of Mathematics and Statistics, University of Otago, PO Box 56,, Dunedin, 9054 New Zealand; 4 Plant Ecology, University of Bayreuth, Universitätstraße 30, 95447 Bayreuth, Germany

## Abstract

There are two prominent and competing hypotheses that disagree about the effect of competition on diversification processes. The first, the bounded hypothesis, suggests that species diversity is limited (bounded) by competition between species for finite ecological niche space. The second, the unbounded hypothesis, proposes that innovations associated with evolution render competition unimportant over macroevolutionary timescales. Here we use phylogenetically structured niche modelling to show that processes consistent with both of these diversification models drive species accumulation in conifers. In agreement with the bounded hypothesis, niche competition constrained diversification, and in line with the unbounded hypothesis, niche evolution and partitioning promoted diversification. We then analyse niche traits to show that these diversification enhancing and inhibiting processes can occur simultaneously on different niche dimensions. Together these results suggest a new hypothesis for lineage diversification based on the multi-dimensional nature of ecological niches that can accommodate both bounded and unbounded evolutionary processes.

## Abstract (web summary)

The degree to which species diversity is bounded by limited niche space is under debate. Here, Larcombe and colleagues show that bounded and unbounded processes contribute more-or-less equally to conifer diversification, and that it may be niche dimensionality that facilitates these opposing forces.

## Introduction

Species diversity has increased dramatically over geological time^1^. Reconstructions using the fossil record are ambiguous about the causes of, and constraints on, this increase^2–4^. One important open question is whether the rate of species accumulation slows as diversity increases, or is independent of diversity^4–6^. The unbounded hypothesis implies that time, and the rate of evolution within clades (monophyletic branches of phylogenies), control diversification and that there is essentially no limit on total diversity^3^. Alternatively, the bounded hypothesis suggests that diversity-dependent processes limit species richness^7^. This limit may be a true carrying capacity, or if extinction is not zero, it is simply the equilibrium between speciation rate and extinction rate^8^. Several mechanisms may cause diversity-dependent dynamics (see ref. ^9^ for a review), and the most widely recognised of these involves competition for limited ecological niche space^7^. Resolving this debate is essential for understanding limits to biodiversity, and why diversity is unevenly distributed in space and time and between clades.

Previous attempts to discriminate between bounded and unbounded diversification have focused on modelling species accumulation as inferred from phylogenies^10,11^ and fossil assemblages^5,6,12^, and to a lesser extent testing how ecological niche evolution impacts diversification^13,14^. The results to date have been inconclusive and often contradictory^2–4,15,16^, indicating that a more nuanced explanation may be required^4,16^. Here we quantify the extent to which both bounded and unbounded processes influence species accumulation in the conifers. Our analysis exploits methodological advances that allow us to infer multi-dimensional physiological niche properties for large suites of species^17,18^. We use these data to discriminate between the distinctive niche characteristics predicted by the bounded and unbounded hypotheses. Specifically we test support for the bounded hypothesis’ prediction that diversification should slow as niche overlap increases within clades^2,8^ and the unbounded hypothesis’ prediction that niche evolution accommodates increasing diversity by allowing the partitioning or expansion of niche space^3,19,20^.

**Fig. 1:**
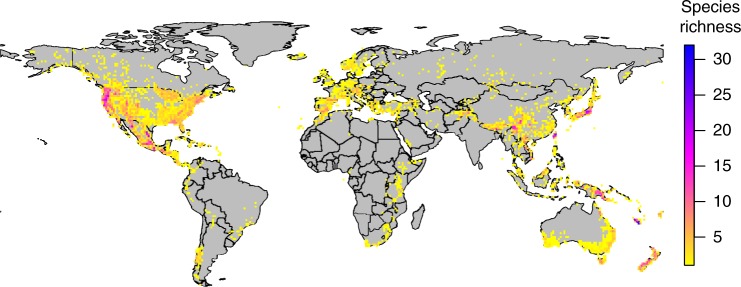
Conifer species richness. Global species richness patterns in 455 conifer species based on cleaned empirical distribution data used here to analyse diversification processes. See Supplementary Fig. 1 for equivalent map of all 600 recognised living conifer species

Conifers are an ecologically important, globally distributed order of plants (Fig. 1; Supplementary Fig. 1) that are ideal for this analysis. This large, well-studied lineage has well-defined clades, excellent distribution data^21^, and is ancient enough (>300 myo^22^) to assess how species accumulate through time. We use distribution data and a process-based species distribution model (SDM) to infer physiological niche parameters for each of 455 living conifer species (75% of extant conifers). The niche parameters are combined with a robust fossil calibrated phylogeny^22^, and interpreted statistically using a range of traditional approaches including correlation analysis and rate through time plots, as well as an a priori conceptual model of how niche and phylogenetic parameters relate to species richness. This conceptual model postulates that species richness can be impacted both directly and/or indirectly by clade age, multivariate niche evolution rate, and two novel metrics: clade niche size and the clade competition index. Clade niche size is the projected potential niche size (number of geographic grid cells occupied by all species in the clade) corrected for clade species number (see Methods). The clade competition index is the product of niche overlap and geographic overlap between species within clades. The parameters of the conceptual model were estimated using phylogenetically constrained Bayesian path analysis. We conduct the analysis at two phylogenetic levels, using 10 large clades and 42 smaller clades. Our analysis shows that bounded and unbounded diversification processes contribute more-or-less equally to diversification in conifers, and indicates that niche dimensionality may be the mechanism by which these opposing forces work together.

## Results and discussion

### Quantifying diversification processes

We produced diversification rate through time plots for the full phylogeny and each of the 10 large clades (Supplementary Fig. 2). This showed a range of patterns including increases, slowdowns, long periods of stasis and multiple rate changes, which is consistent with both bounded and unbound processes influencing diversification in conifers^9,23,24^. However, it has been shown that a number of factors may confound patterns of diversification derived from phylogenies in this way, and they are likely to be especially problematic in old lineages with unobservable extinction^2,23,24^. Therefore given that conifers are an ancient lineage (>300 million years old) that are believed to have been strongly influenced by Cenozoic extinctions^25^, we pursued other forms of evidence to identify diversification dynamics in this group.

**Fig. 2:**
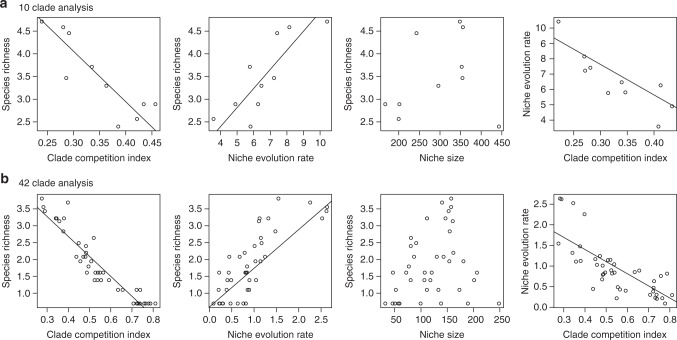
Associations between species richness and diversification metrics. Scatter plots between clade species richness and selected clade metrics for two divisions of the conifer phylogeny into **a** 10 large clades and **b** 42 smaller clades. Straight lines indicate significant linear effects detected using phylogenetic generalized least squares (PGLS) regressions. The presence of multiple correlations made interpretation difficult; for this reason, we performed a path analysis (Fig. 3)

To begin, we estimated the extent of correlations between indices of diversification, species competition, species richness and niche size. These analyses suggest that both bounded and unbounded processes influenced diversification (Fig. 2). In line with bounded diversification, the clade competition index was negatively related to species richness, and, as predicted by the unbounded hypothesis, niche evolution was positively correlated with species richness (Fig. 2). There was no clear relationship between clade niche size and species richness, suggesting that niche partitioning is an important diversification process (Fig. 2). That is, if speciation was largely occurring as a result of niche expansion—where adaptation facilitates new species accessing new ecological space—we would predict a positive relationship between clade niche size and species richness because new species expand the total clade niche size. Conversely, if speciation is occurring via specialization and the division of existing clade niche space (i.e., niche partitioning) we would predict no relationship between clade niche size and species richness because adding new species does not expand total clade niche size. The correlations further suggested a negative relationship between the niche evolution rate and clade competition index. Unfortunately, these simple correlation analyses cannot elucidate the relative effects nor the role of indirect effects of the factors on clade species richness. For these reasons we performed a phylogenetically constrained path analysis.

**Fig. 3:**
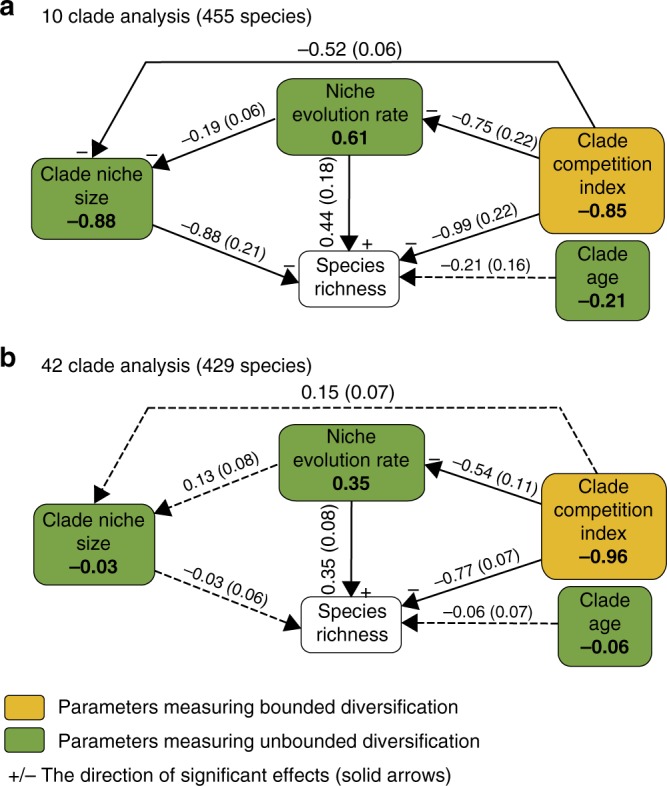
Path analysis of variation in conifer species richness. Bayesian path analysis showing the relative effects of niche and phylogenetic parameters on clade species richness for 455 conifer species in **a** 10 large clades and **b** 42 smaller clades. Total effect size is shown in bold, while direct effects and their standard deviation are shown along the vertices. Solid lines indicate significant effects (95% credible intervals not including zero)

**Fig. 4:**
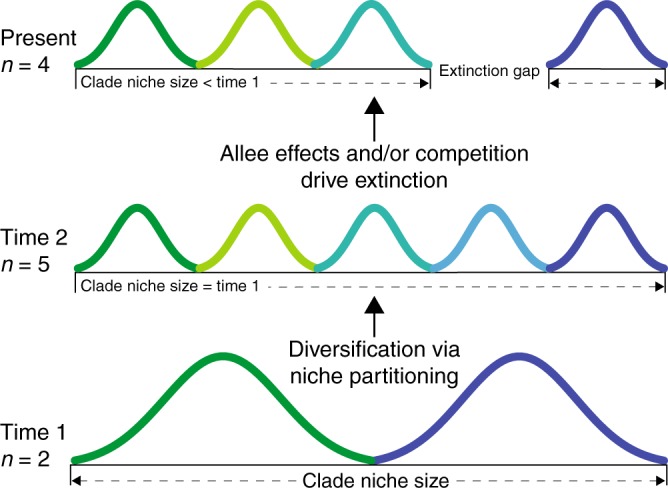
Extinction driven reduction in clade niche size. Example of how niche partitioning combined with extinction associated with allee effects and/or competition, can result in a negative relationship between clade niche size and species richness as found in Fig. 3a. Different coloured curves represent species

The path analyses revealed that diversification in conifers was influenced in almost equal measure by bounded and unbounded processes (Fig. 3). In line with the bounded hypothesis, competition with relatives (clade competition index) had a strong negative effect on species richness, which suggests that available niche space can limit species accumulation. This effect was strong in both the 10 (*r* = −0.85) and 42 (*r* = −0.96) clade analyses. Support for the unbounded hypothesis was evidenced by our finding that niche evolution rate contributed positively to species richness, suggesting that higher niche evolution rates within clades allow more species to accumulate. This effect was stronger in the 10 clade analysis (*r* = 0.61) than in the 42 clade analysis (*r* = 0.35). Furthermore, we found that clade niche size had neutral (42 clade analysis) or negative (10 clade analysis) influence on species richness, again suggesting that niche partitioning constitutes the main mode of niche evolution in conifers. The negative effect of clade niche size (10 clade analysis) is somewhat counter-intuitive since it suggests that clades with smaller niche volumes accommodate more species. However, this pattern is consistent with niche partitioning accompanied by allee effects and/or competition^8,26^ driving random extinction processes that lead to a reduction in clade niche size as postulated in Fig. 4. In fact the significant direct effects of competition (*r* = −0.52) and niche evolution (*r* = −0.19) on clade niche size, and relatively strong negative effect of clade age (*r* = −0.21) on species richness (Fig. 3a), are consistent with such competition driven extinction processes unfolding through time^8^. The lack of evidence for this causal pathway in the 42 clade analysis probably reflects the much younger average clade age (17 my compared with 112 my), and smaller clade sizes, which mean that partitioning and extinction processes (Fig. 4) will be less frequent and therefore more difficult to detect. This interpretation is consistent with previous work suggesting extinction played a pivotal role in the diversification of conifer clades in the Cenozoic, while younger clades are primarily shaped by recent speciation^25^.

We note that our clade competition index under-estimates competition because it may not capture all potential competitive interactions. Our measure quantifies expected competition between members of a clade based on overlap in geographic space and niche space (see Methods). It is an underestimate because, although competition is likely to be most intense between close relatives (i.e., members of the same clade), competitive interactions with more distantly related species are also likely and not captured by our metric^27–29^. Incorporating competition with distantly related species, although possible, would require additional data and necessitate additional assumptions. It is also possible that our clade competition index fails to detect some forms of competition that might constrain diversification rates. For example, it is possible that competition between ecologically similar species may prevent them from becoming sympatric as has been reported in some bird lineages^30,31^. Such processes could limit range expansion and potentially reduce diversification rates if range expansion increases the likelihood of diversification—for example by increasing the probability of allopatric speciation^9^.

Although much previous work has favoured either bounded^2,10,14,28^ or unbounded^3,32,33^ processes driving diversification, our results are consistent with observational^12,19^, theoretical^16^ and modelling^4,12^ work, which suggests that both bounded and unbounded processes influence diversification. For example, much of the empirical evidence is consistent with diversification slowing, rather than reaching an asymptote^16,19^. This led Cornell^16^ to propose the “damped increase” hypothesis, which in line with our results, suggests that competition induced by niche filling reduces diversification rate, while specialisation or new ecological opportunities counteract this effect^16^. Others have extended these ideas to show that the incongruity between strict bounded and unbounded views could be overcome by allowing diversity-accumulation-models to vary between periods of either bounded or unbounded diversification^4^. These studies do not, however, provide a population/species level mechanism that could drive shifts in diversification processes^4^.

### Niche dimensionality and diversification

To address this mechanistic basis, we examined whether niche dimensionality can drive variation in diversification processes^4,34^. We used a range of statistical procedures to determine if variation exists in the evolutionary flexibility of niche traits at three levels: (1) across the phylogeny; (2) within clades; and (3) between clades. Across the full phylogeny we found variation in the level of conservatism (phylogenetic signal) between traits (with Pagel’s *λ* values ranging from <0.01 to 0.42; Table 1), suggesting variation in the evolutionary flexibility of niche dimensions. This variation between traits was also evident within clades, for example in Clade 7, *Pinus* (Table 2). In fact, mixed effects modelling show that significant variation exists in evolutionary rate between traits after accounting for random variation between clades (*trait*: *F*_10,90_ = 62.5, *P* = <0.0001), suggesting that trait evolution rates do, on average, vary within clades.

**Table 1:** Phylogenetic signal across the full phylogeny

Niche trait	*λ*	P(*λ*)	K	P(K)
Soil moist N uptake (3)	0.144	<0.001	0.019	0.001
Max temp growth (4)	0.000	1	0.016	0.039
Min temp growth (3)	0.092	<0.001	0.016	0.115
Soil moist N uptake (2)	0.444	<0.001	0.023	0.001
Mean temp growth (2)	0.205	<0.001	0.020	0.001
Soil moist growth (2)	0.362	<0.001	0.021	0.001
Radiation growth (2)	0.054	0.064	0.018	0.005
Min temp growth (2)	0.415	<0.001	0.024	0.001
N soil growth (1)	0.158	<0.001	0.019	0.004
N soil growth (2)	0.132	<0.001	0.017	0.012
Mean temp N uptake (2)	0.215	<0.001	0.021	0.001

Phylogenetic signal in the 11 key niche traits based on a conifer phylogeny covering 455 species and estimated using Pagel’s *λ* and Blomberg’s K. The *p*-value (P) for *λ* is estimated using the likelihood ratio test. The *p*-value for K is estimated from a randomization test based on 1000 simulations of the data. The numbers in parentheses indicate the position of the specific trait in the growth or resource acquisition function. For example Soil moist N uptake (3), is the point at which increasing soil moisture, starts to limit nitrogen (N) uptake, i.e. when water logging limits N uptake. See Results and Discussion for details

**Table 2:** Phylogenetic signal within Clade 7 (*Pinus*)

Niche trait	*λ*	P(*λ*)	K	P(K)
Soil moist N uptake (3)	0.569	0.003	0.080	0.005
Max temp growth (4)	0.000	1.000	0.076	0.011
Min temp growth (3)	0.000	1.000	0.058	0.286
Soil moist N uptake (2)	0.623	0.026	0.097	0.001
Mean temp growth (2)	0.167	0.051	0.092	0.001
Soil moist growth (2)	0.609	0.033	0.079	0.003
Radiation growth (2)	0.000	1.000	0.079	0.005
Min temp growth (2)	0.104	0.208	0.072	0.015
N soil growth (1)	0.000	1.000	0.067	0.076
N soil growth (2)	0.000	1.000	0.062	0.159
Mean temp N uptake (2)	0.004	0.894	0.077	0.004

Phylogenetic signal in the phylogeny of 111 species of *Pinus* for 11 key niche traits, estimated using Pagel’s *λ* and Blomberg’s K. The *p*-value (P) for *λ* is estimated using the likelihood ratio test. The *p*-value for K is estimated from a randomization test based on 1000 simulations of the data. The numbers in parentheses are as Table 1

**Fig. 5:**
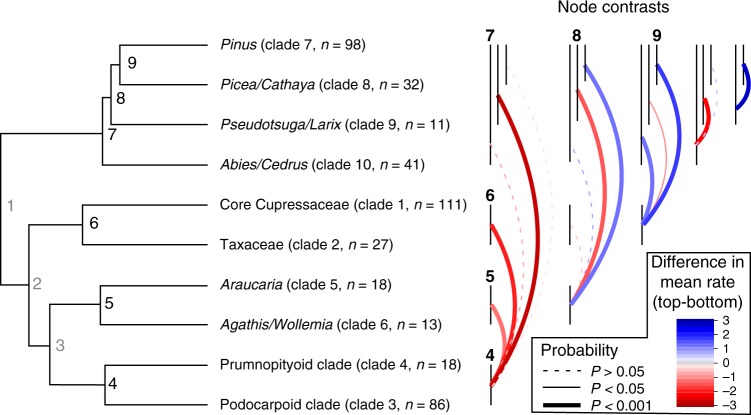
Phylogenetic independent contrasts of niche evolution rate. Left: clade-level conifer phylogeny showing terminal nodes (4–9) in bold black, internal nodes (1–3) in grey (not consider in the contrasts). Taxonomic information about the groups, as well as their clade number and species richness (Clade #, *n* = species richness) are given at the tips. Right: Contrasts between the terminal nodes. The vertical bars correspond to the terminal nodes (labelled once in bold to match those on the tree), and the coloured arcs show the difference in mean niche evolution rate between the nodes (calculated by subtracting the mean of the top node from the mean of the bottom node). For example the red line joining nodes 5 and 4 indicates that the niche evolution rate at node 5 is 1.3 lower than node 4. Significance is indicated by line thickness and type. The pattern that emerges is that the nodes which give rise to clades with high species richness (e.g., nodes 4 and 9) have significantly higher niche evolution rates than nodes giving rise to clades with low species richness (with the exception of node 6, see Results and Discussion). For further details on the contrasts see Supplementary Table 1

We also found that the evolution rate of traits varies between clades, for example, Fig. 5 summarises how the evolution rate of traits varies across the clade-level phylogeny after accounting for non-independence associated with phylogenetic relationships (using phylogenetic independent contrasts^35^). This analysis indicates that trait evolution rate varies significantly across the terminal nodes of the clade-tree (node: *F*_8,80_ = 6.4, *p* < 0.0001). Looking at the terminal nodes is interesting because it provides inference regarding the descendent clades, and Fig. 5 shows that high rates of trait evolution are often associated with increases in diversity, and vice versa. For example, nodes that give rise to relatively high diversity clades (e.g., nodes 4, 7 and 9; Fig. 5) tend to have have significantly higher trait evolution rates than nodes that give rise to lower diversity clades (i.e., nodes 5 and 8; Fig. 5). The only exception to this pattern is node 6, which is parent to the high diversity Clade 1 (*n* = 111), and has a relatively low trait evolution rate (Fig. 5). Interestingly, Clade 1 also has the lowest clade competition index of any clade in our analysis, possibly suggesting that in the absence of strong competition, diversification has advanced without a parallel increase in trait evolutionary rates. Allopatric speciation in an ecologically specialised, and well dispersed linage might explain this type of pattern. The most diverse genus in Clade 1, *Juniperus*, is unusual among conifers in its preference for relatively arid, warm climates and calcareous soils, furthermore the evolution of “berry-like” fruits is thought have driven extensive dispersal and allopatric speciation in the genus^36^. Together, the above results imply that trait evolutionary rates vary within and between conifer clades, and in combination with competitive interactions this variation can explain shifts in clade-level diversity.

**Fig. 6:**
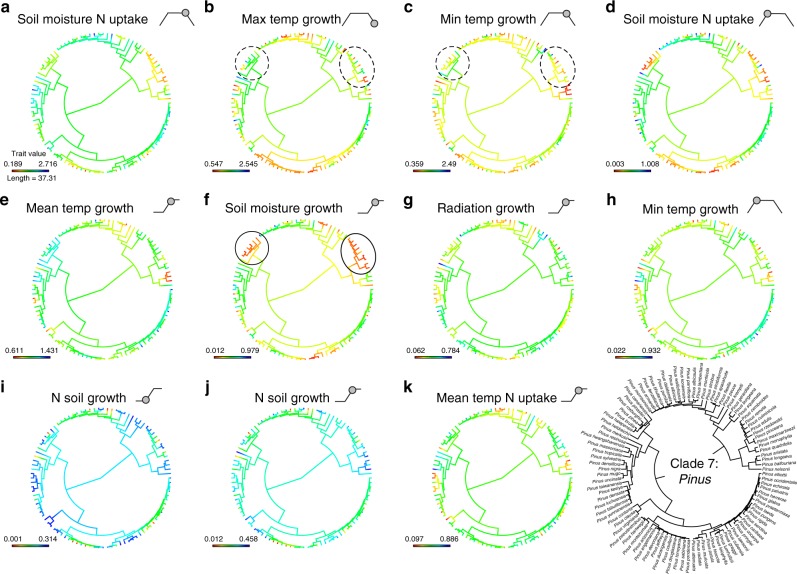
Niche dimensionality in *Pinus*. Phylogenies of Clade 7 (*Pinus*) showing ancestral state reconstructions of the 11 most important niche dimensions in order of importance (**a**–**k**). The bottom right panel shows the same phylogeny with species names. Sub-clades within *Pinus* with conservative (solid ellipse) and labile (dashed ellipse) niche dimensions are highlighted and discussed in the text (see Results and Discussion). The filled circle on trapezoid and step diagrams beside the trait names, show how the trait relates to the modelled growth or resource acquisition function. For example, (**a**) is the point at which soil moisture causes a reduction in N uptake, that is, when waterlogging reduces N uptake

Such variation in the evolutionary flexibility of traits and competition between species within clades may accommodate the operation of both bounded and unbounded processes. This can be seen more clearly by focussing attention on single clades. For example, in Clade 7 (*Pinus*, Fig. 6), the effect of soil moisture on growth (Fig. 6f) is highly conserved in the sub-clades highlighted with solid ellipses, suggesting that interspecific competition is likely to be high along this niche dimension in these sub-clades. However, these same sub-clades are labile in terms of their temperature requirements for growth (traits b and c, highlighted with dashed ellipses in Fig. 6b, c), indicating that evolution and specialisation are possible along these niche dimensions (Fig. 6). Analogous patterns can be seen in the other *Pinus* sub-clades (Fig. 6) and the other clades (Supplementary Fig. 3). Population level studies investigating how individual traits respond under direct interspecific competition in actively diversifying linages are needed to help clarify how these processes operate at the ecological level.

By considering the multi-dimensional nature of niche evolution, we have shown how bounded and unbounded diversification processes may simultaneously control diversification rates. Niche dimensionality has long been thought to promote diversity by partitioning resources and facilitating coexistence^37^, and there is considerable empirical support for this hypothesis^34^. Most previous assessments of how niche characteristics impact macro-diversification have used low dimensional proxies of the niche such as body size^14^ or climatic range^13^. In contrast, our assessment of multiple, physiological niche traits, reveals that both diversity-limiting competition, and diversity-promoting evolution may operate concurrently. At the population level these processes are likely to be separated in space and/or time—in line with models by McPeek^38^ and Marshall and Quental^4^, respectively. For example, populations along environmental gradients could experience variation in the opportunity for specialisation or niche expansion along some niche dimensions but experience competition along other niche dimensions^38^. Similarly, changes in the environment could induce temporal variation in selection pressure that affects the interplay between conservative and labile niche traits^4^.

In summary, we have identified how processes that define the niche geometry of conifer clades can jointly promote and constrain diversification. Our results confirm that the contrasting processes that underpin bounded and unbounded diversification have both operated during the evolution of a major lineage. Our study thereby provides an analysis framework for a new multi-dimensional-niche hypothesis that unifies the bounded and unbounded hypotheses^4,12,16,19^.

## Methods

### Data acquisition and preparation

Geo-referenced collection data for all conifer species were extracted from the Global Biodiversity Information Facility (www.gbif.org). These data were supplemented by published species records not in GBIF from^39–44^. Climate estimates were made for each point record, using Worldclim^45^. Data was cleaned manually by firstly eliminating duplicate records, then for consistency with species distribution descriptions^39^, and then by comparing Worldclim estimates of altitude, with the altitudes provided with each site record. Where Worldclim altitudes were inconsistent with the altitude in species descriptions by more than 300 m, we replaced these records with estimates from nearby sites with altitudes consistent with the descriptions.

### Estimating physiological niche traits

We estimated the physiological niche traits of the study species using a physiologically-based approach to species distribution modelling^17^. This method uses the Thornley transport resistance (TTR) model of plant growth^46^ to estimate the niche traits that match the observed distribution of species. The TTR model^46^, is an ordinary differential equation that models how plant growth is influenced by carbon uptake, nitrogen uptake, and the allocation of carbon and nitrogen between roots and shoots. It explicitly separates the physiological processes of resource uptake from biomass growth. The implementation by Higgins et al.^17^ relates the uptake and growth processes to environmental forcing variables. Specifically, the model considers how carbon uptake might be limited by temperature, soil moisture, solar radiation and shoot nitrogen; nitrogen uptake might be limited by temperature, soil moisture and soil nitrogen; and growth and respiration loss might be influenced by temperature. The model runs on a monthly time step, which allows it to explicitly consider how seasonal fluctuations in the forcing variables interactively influence plant resource uptake and growth. Higgins et al.^17^ provides a full description of the model and its assumptions.

We use the cleaned presence dataset described above to identify locations where the species occur. A variety of methods for simulating absence points (often called pseudoabsence points) are available, but the method adopted is regarded as a relatively small source of error^47^. Our method balances the number of presence and absence points and stratifies the selection of absence points by environment type. To define environment types we use a partitioning algorithm *clara*^48^ to classify the TTR environmental forcing variables into 25 environmental zones. We further restricted the selection of absence points to the zoological realm(s) where the species occurs and to distances >0.25  degrees from the presence points. This approach helps ensure that a representative range of environmental zones are included in the absence samples and that they are selected within a dispersal zone that is potentially reachable on an ecological time scale (i.e., the zoological realms).

The model predicts the potential biomass of an individual plant as a function of the environmental forcing variables at a site. Following the work of Higgins et al.^17^, we assume that *p*_i_, the probability of a species occurring at site *i*, is described by the complementary loglog of the modelled plant biomass at site *i* and that the likelihood of observing the presence–absence data (*y*_*i*_) at site *i* is described by the Bernoulli distribution. To estimate the parameters, we used the differential evolution optimization algorithm^49^ to find the set of parameters that maximizes this likelihood over all sites. The model fits were evaluated by examining the confusion matrix (a matrix comparing the number of true positives, true negative, false positives and false negatives), with particular weight given to the false negative rate, i.e., instances where the model predicts the species to be absent, but it is actually present (Supplementary Data 1).

Like most species distribution modelling techniques, our analysis predicts the potential niche of a species. In most situations biotic interactions and dispersal limitations will prevent species occupying the full extent of their potential niche. With this in mind we restrict projection of potential species ranges to the subset of environmental zones (see above) present in each species’ occurrence data; this prevents predictions beyond the data domain used for estimating the model parameters. We calculated the niche size of species in two ways: (1) projecting species ranges for the world, and (2) using a resampled dataset that assumes that the worlds environmental zones are equally common. This second method corrects for any bias in projected range size introduced by variation in the extent of different environmental zones, but maintains the covariance structure of the environmental data^50^. To create a dataset where each environmental zone is equally common, we created a resampled dataset of the environmental data. We again use *clara* to classify the global TTR input data into 50 environmental zones. We then sampled a finite number (1000 in our case) of locations from each of 50 environmental zones, which produces an environmental dataset where each environment zone is equally represented. We projected the range sizes of species in this resampled environmental space. Analyses conducted using geographic locations and resampled locations yielded very similar results. The analysis based on resampled locations is presented in the main manuscript while the analysis based on geographic locations is available in Supplementary Fig. 4.

### Phylogenetic methods

We used the fossil calibrated conifer phylogeny of Leslie et al.^22^, which is based on two chloroplast genes and two nuclear genes. We pruned this 487 species tree to match the 455 species for which we had good distributional data. Although a clade is any monophyletic group in a phylogeny, the ability to detect effects in clade-wise analysis will be in part reliant on having enough variation in clade size^51^. Therefore we developed two clade classifications. The first inclusive division is based on tree topology at deeper well supported nodes, and it aimed to retain major taxonomic groups such as *Pinus*, resulting in 10 clades (Supplementary Data 1). The second lower division is based on a time-slice approach at Eocene/Oligocene boundary (33.9 ma). Using the tree topology closer to the tips than this becomes more difficult. This second approach produced 68 clades, 28 of which included a single species. These single species were dropped from the analysis, leaving 42 clades and 429 species in the second analysis (Supplementary Data 1). We recognize that removing single species clades might bias rate estimates because these are the clades with the lowest diversification. However, the dataset still covers a wide range of clade species richness (2–45 species), and meaningful estimates for single species cannot be calculated for most subsequent metrics used in our analysis (e.g., niche evolution rate, clade niche overlap, clade geographic overlap etc.). Furthermore, this potential bias only affects the 42 clade analysis and the general agreement between the 10 and 42 clade analyses (see Results and Discussion) suggests that any effect is inconsequential.

We produced diversification rate through time plots using BAMM (Bayesian analysis of macroevolutionary mixtures). BAMM was run on the full tree of 455 species with following parameter settings: the sampling fraction was set at 0.762; the priors were estimated from the tree using setBAMMpriors in BAMMtools^52^ in R, the expected number of shifts was one, the lambda initial prior was 12.414, the lambda shift prior was 0.003414, the mu initial prior was 12.414 and the lambda time variable prior was 1; MCMC was run for 2,000,000 generations, write frequency was 2000, print frequency was 100, and the acceptance rate was 10; all other settings were set to the BAMM defaults. Clade-level BAMM runs were done using clade-level phylogenies pruned from the full phylogeny, sampling fractions adjust to reflect exact clade coverage, and priors were adjusted using setBAMMpriors. Rate through time plots with confidence shading were produced in BAMMtools for the full tree and each of the 10 clades separately (Supplementary Fig. 2).

### Clade-level metrics

For each clade we calculate the following metrics: age; niche size (number of geographic grid cells occupied by all species in the clade) is the projected potential niche size; niche evolution rate; and clade competition index. The crown age of the clade was calculated directly from the tree using the branching time function in APE^53,54^. When assessing niche size, we needed to control for  the number of species in the clade. In the 10 clade analysis, the smallest clade contained 12 species. Instead of using the direct niche size of each of a clade, we instead randomly subsampled subsets of 12 species from each clade, computing the niche size for each subsample, and taking the mean of these values. The resampling was repeated 10,000 times. In the 42 clade analysis we used the same procedure, except that subsamples of size 2 were used.

The calculation of niche evolution rate involves using a multivariate model. The TTR species distribution model estimates 24 parameters associated with plant growth (see above). For this reason we first extracted the most informative of the 24 niche parameters for the analysis, specifically we used phylogenetically corrected principal components analysis (PCA)^55^ to identify which model parameters had the most influence on shaping niche space in our dataset. PC 1 to 8 explained over 94 percent of the variation in the dataset. The most influential parameters were identified based on the eigenvector loadings >0.3, and vector plots were used to exclude correlated parameters. This procedure identified 11 parameters (Figs 5, 6) which were ranked in order of importance by summing the effect of each trait on each PC weighted by the proportion of the variance explained by that PC. For the 10 clade analysis, these 11 parameters were fit together in a multivariate Brownian motion (BM) model of evolution in OUCH^56^. In the 42 clade analysis, because some clades had only two species there were insufficient degrees of freedom to use a multivariate model, and a univariate Brownian motion (BM) model of evolution was fitted using the most important parameter (the effect of soil moisture on N uptake^3^; see Figs 5, 6). Following^13^, the trait evolution model was used to calculate the variance–covariance matrix for the traits in each clade. The diagonal elements of this trait matrix represent the phylogenetic rate of character evolution which were summed to provide a multivariate (or univariate) rate parameter for each clade—the niche evolution rate^13^.

The bounded hypothesis proposes that competition plays a key role in limiting diversification. Competition is likely to be most intense between close relatives due to similar physiological requirements (or niches) wherever species co-occur^8^. To estimate competition, we produce a metric which summarises the degree of expected niche overlap and observed geographic overlap between species within clades. Schoener’s index^57^ of niche overlap was estimated for each pair of species from the projected species distributions (i.e., the potential niche of the species) in SPAA^58^, and the subsequent matrix was rescaled so the values range between 0 and 1. For each pair of species we computed the the average distance between each geo-referenced occurrence record for one species and each geo-referenced occurrence record in the other. These values were also normalised over all pairs of species to produce a matrix of values between 0 and 1. We subtracted each value from 1 to give measures of species overlap. The competition index for two species is defined to be the product of the niche overlap and the measure of geographic overlap. The “clade competition index” for a clade is defined as the average of the competition indices between all species in the clade.

This formulation of the clade competition index ensures that, if species are randomly permuted, the expected value of the index for a clade is simply equal to the mean competition index between all pairs of species (by the linearity of expectation). Hence, the expected clade competition index is independent of clade size. We further verified the lack of bias by simulation. We randomly shuffled the species names at the tips of the phylogeny, to produce 10 clades of the same size as those in our analysis, but with a random compliment of species. We then calculated the clade competition index for each randomised clade as above and stored this result. This process was repeated 10,000 times. The average clade competition score (based on the 1000 replicates) was then plotted against species richness for comparison with the empirical data (see Supplementary Fig. 5). We used least squares regression to test the relationship between clade species richness and the randomised clade competition index, with the expectation that any bias in the metric would result in a significant deviation from zero.

### Regression modelling

Correlations were investigated between species richness (log-transformed) and the clade competition index, niche evolution rate and clade niche size as well as between the niche evolution rate and clade competition index. Phylogenetic generalized least squares (PGLS) regression models were used to look for significant correlations, with the clade competition index and niche evolution rate square root transformed to meet the assumptions of normality.

We developed an a priori conceptual model (Fig. 3) to estimate the relative effects of clade niche size, niche evolution rate, clade age and the clade competition index on species richness. The unbounded model predicts that specific evolutionary characteristics, controlled by phylogenetic niche conservatism, lead to clade-specific diversification rates. This has two consequences: (1) when the effect of diversification rate is factored out older clades will have more species than younger clades; and (2) positive diversification will involve niche evolution that manifests as either the expansion or partitioning of clade niche space as species accumulate. In line with these predictions our model allows: (1) clade age to directly influence species richness; and (2) niche evolution rate to influence species richness both directly, and indirectly, via its effect on clade niche size, with the direct relationship between clade niche size and species richness indicating the mode of niche evolution (expansion or partitioning). Conversely, the bounded diversity model predicts that competition for limited resources places a limit on species number. It has long been recognised that competition is likely to be most intense between close relatives, because the ecological requirements of relatives are likely to be similar due to phylogenetic niche conservatism. Our clade competition index quantifies competition between the species within a clade. Therefore we allow the clade competition index to directly effect species richness, however, because the clade competition index quantifies interactions between niches, it is also allowed to indirectly influence species richness via its effect on the niche evolution rate, and clade niche size.

We used Bayesian path analysis to calculate the effects in the path diagram (Fig. 3), while accounting for non-independence associated with phylogenetic relationships^59^. The total effect of each model parameter on the response variable (species richness) was calculated from the direct and indirect effects following Schumacker and Lomax^60^. All model parameters were normalised and centred to a mean of zero and constant standard deviation. Following Rabosky et al.^61^, we use relative log-transformed species richness. For each analysis (10 and 42 clade), the full phylogenetic tree was collapsed to the clade level, and the inverse of the variance–covariance matrix from this clade-tree was used to explicitly correct for the phylogenetic dependencies between clades. Modelling was undertaken using JAGS^62^ running three chains for 15,000 iterations, after a burnin of 25,000, and thinning the chains to every fifth sample. Normal uniformed priors we used for the path effects. The package coda^63^ was used to produce trace plots for diagnosing convergence.

### Niche trait analysis

We used a phylogenetic trait analysis to quantify the evolution of individual niche dimensions at the level of the full phylogeny and within clades. This analysis focused on the 11 niche dimensions identified above. Phylogenetic signal across the full phylogeny and in detail for clade 7 (*pinus*), was estimated using Pagel’s *λ*^64^, with significance assessed using likelihood ratio tests, and Blomberg’s K^65^, with simulations to assess significance, in PHYTOOLS^55^. The PHYTOOLS function “contMap” was used to produce ancestral state reconstructions for each of the 11 most important niche traits. We also made clade-level ancestral reconstructions of the 11 main niche dimensions for the 10 large clades to visually assess variation in the conservation of niche dimensions within clades (Fig. 6; Supplementary Fig. 3).

A second round of niche evolution modelling focused on estimating the evolution rate of 11 primary niche dimensions independently for each clade in the 10 clade analysis. This was done as above, except single variate BM models were fitted in OUCH rather than multivariate models. These clade-level trait evolution rates were used in two subsequent analyses. Firstly, in order to test for clade-level variation in evolution rate between traits, we fitted linear mixed models treating log-transformed evolution rate as the dependent variable, trait as a fixed effect and clade as a random effect using the R package nlme^66^. Secondly, in order to account for non-independence associated with the phylogenetic relationships, we rescaled the log-transformed evolution rate for each trait using phylogenetic independent contrasts in the R package ape^54^. This procedure produced phylogenetic independent estimates of the mean trait evolution rate for each node in the 10 clade phylogeny (Fig. 5). We used analysis of variance to determine if PIC evolution rate varied between different nodes, and computed contrasts between all terminal nodes using the Tukey honest significant difference (Fig. 5; Supplementary Table 1).

### Code availability

Computer code that supports the findings of this study are available from the corresponding author upon request.

## Electronic supplementary material

Supplementary Information

Description of Additional Supplementary Files

Supplementary Data 1

## Data Availability

The data that support the findings of this study are available from the corresponding author upon request.
