# Supplementary material for: The dimensionality of niche space allows bounded and unbounded processes to jointly influence diversification
Source: Nat Commun. 2018 Oct 15;9:4258. doi: 10.1038/s41467-018-06732-x (PMC6189034; doi:10.1038/s41467-018-06732-x)
Supplement: Supplementary file 1 — Supplementary Information [file 41467_2018_6732_MOESM1_ESM.pdf]

Supplementary Information

The dimensionality of niche space allows bounded and unbounded processes to jointly influence diversification

Larcombe et al. 2018.

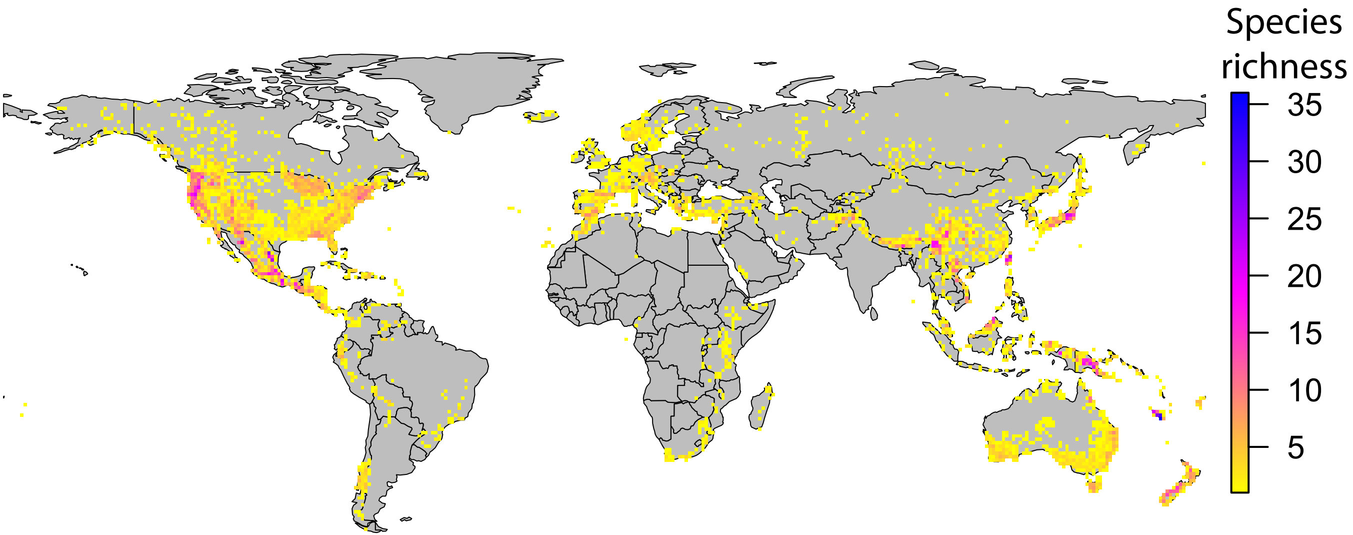

Supplementary Figure 1. Global species richness patterns in all 600 living conifer species based on cleaned empirical distribution data.

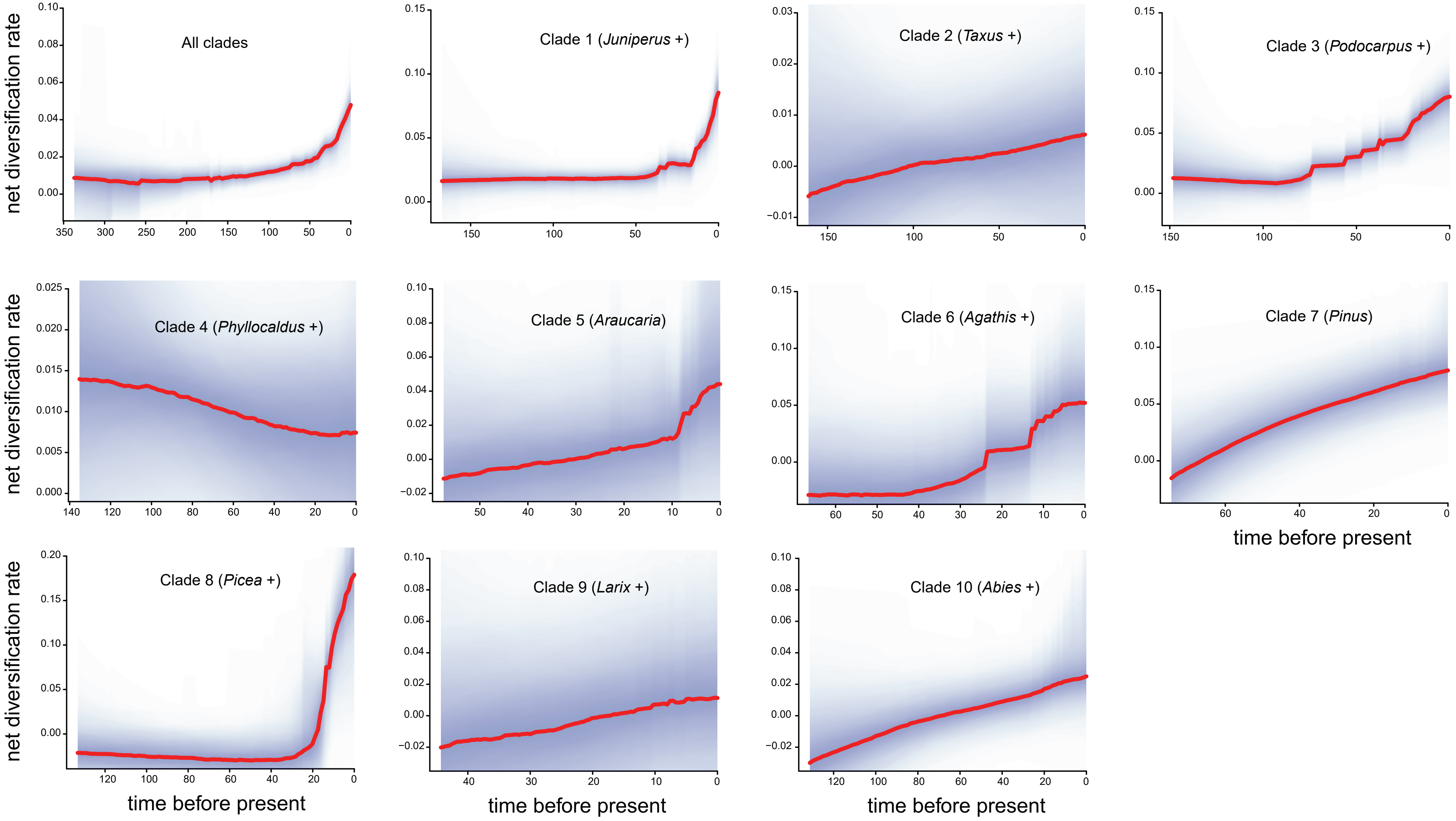

Supplementary Figure 2. Diversification rate through time plots for the phylogeny with 455 conifer species and each of 10 clades independently. Shading shows the confidence of the evolutionary rate reconstruction for any given time point based on BAMM (Bayesian Analysis of Macroevolutionary Mixtures) analysis.

Supplementary Figure 3 [caption]. The next 10 pages (Supplementary Fig. 3a-j) each show phylogenies from one of Clades 1:10. In each case ancestral state reconstructions of the 11 most important niche dimensions are given in order of importance (panel's a-k) and in the bottom right the same phylogeny is given with species names (the position of important genera/species are given where the species names are illegible). The trapezoid and logistic diagrams following the trait names show the position of the trait in modelled growth or resource acquisition function. For example, a) shows the point at which the soil moisture effect begins to reduce N uptake, i.e. when water logging starts to reduce N uptake.

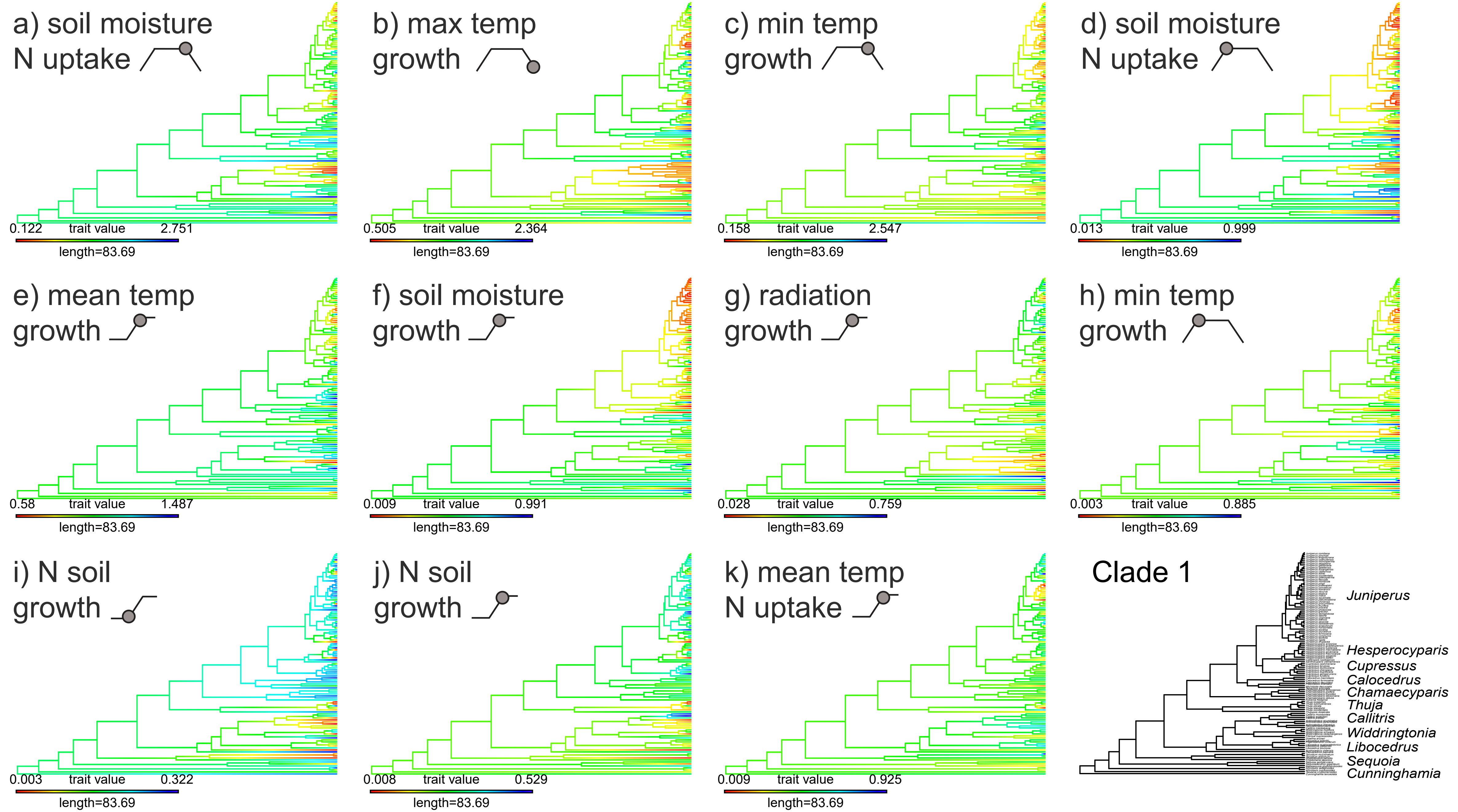

Supplementary Figure 3a

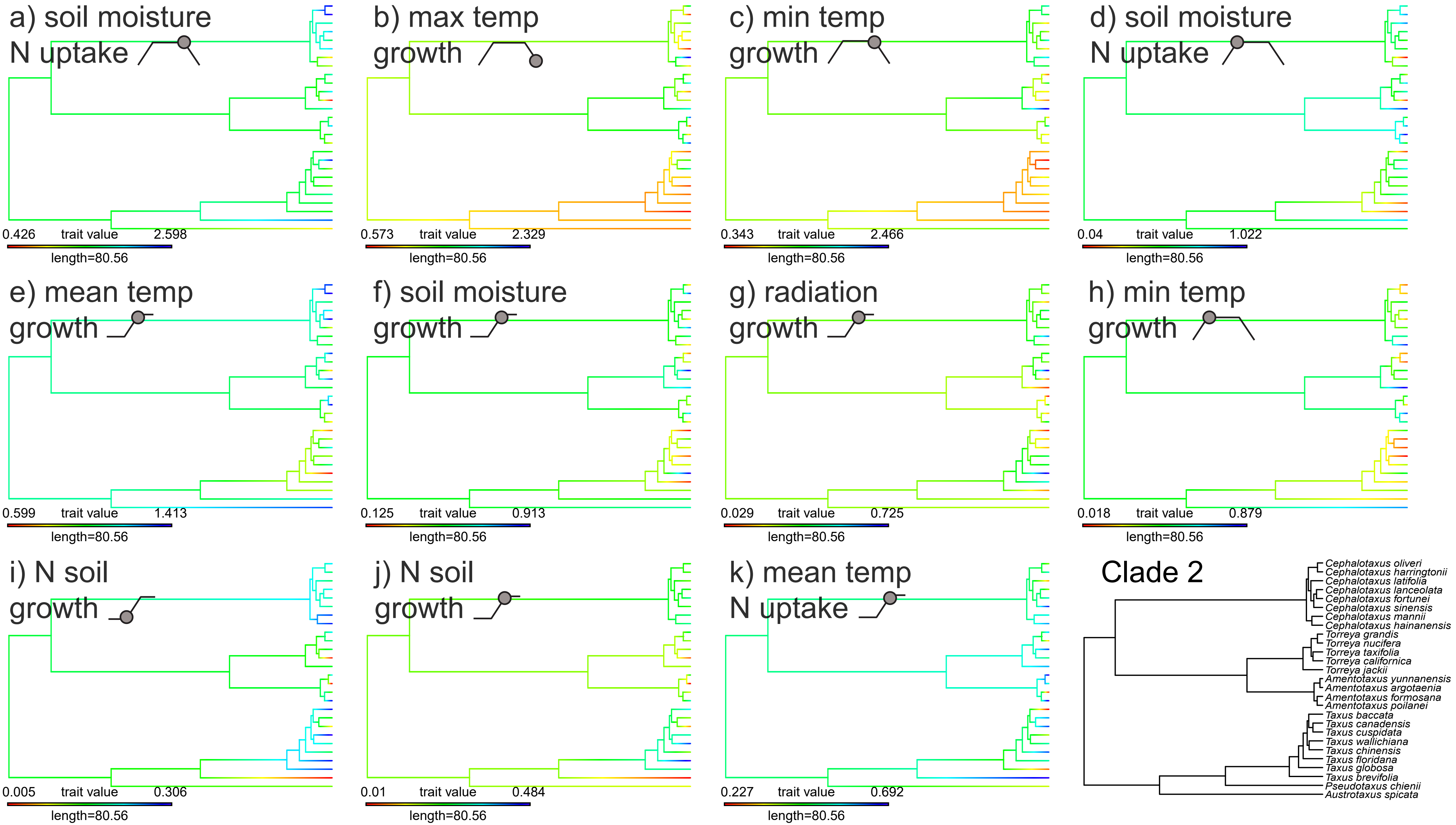

Supplementary Figure 3b

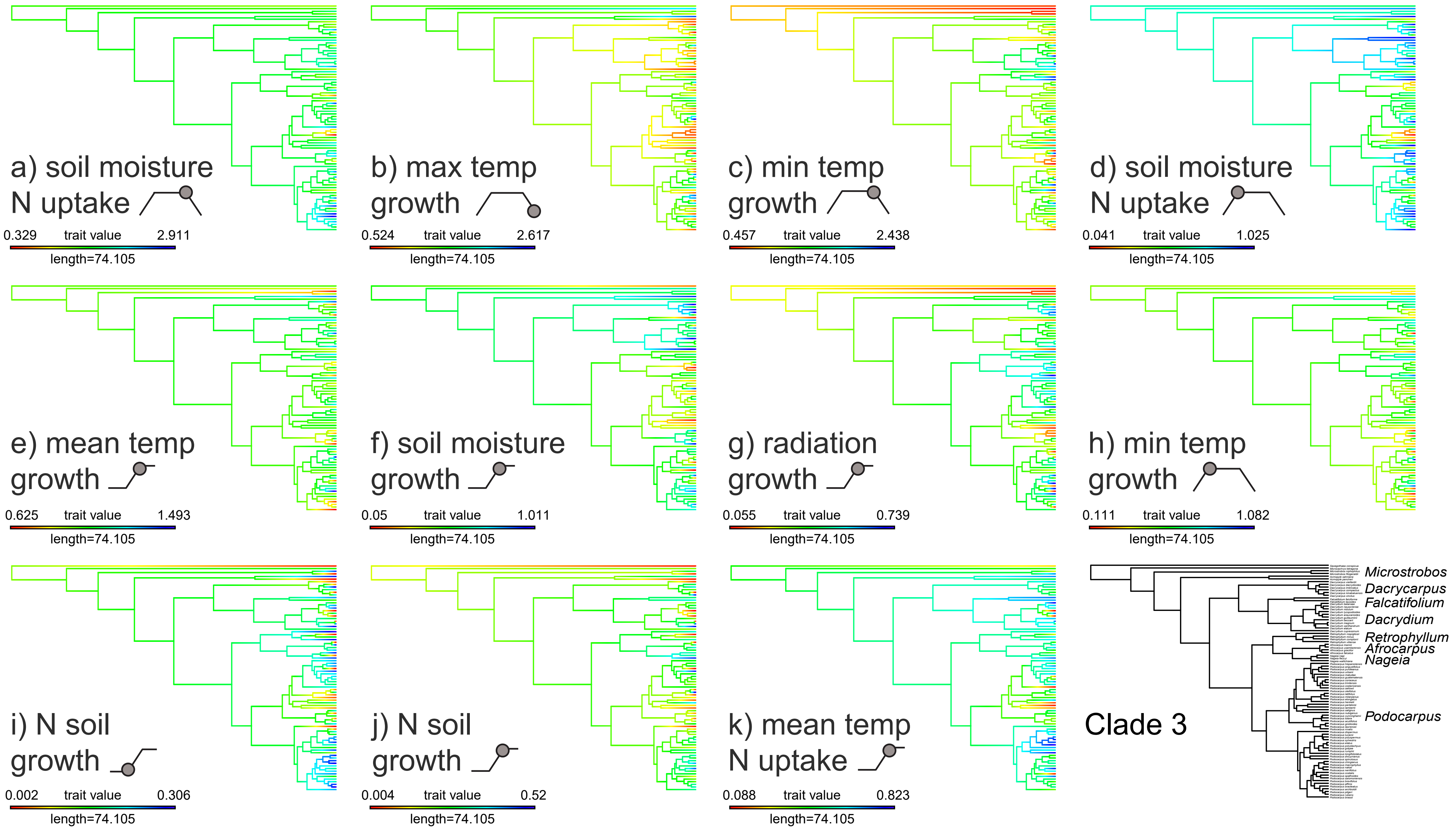

Supplementary Figure 3c

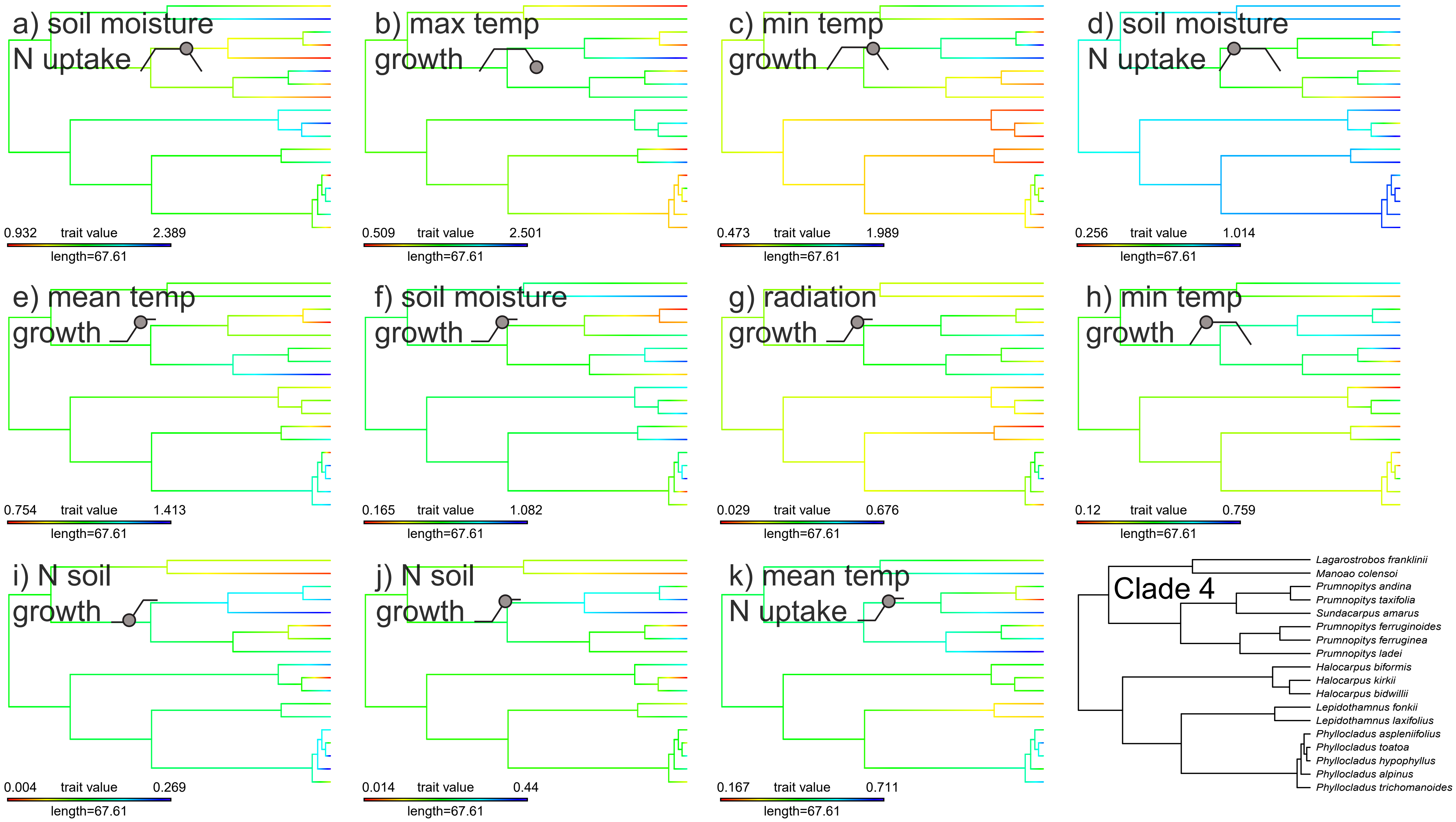

Supplementary Figure 3d

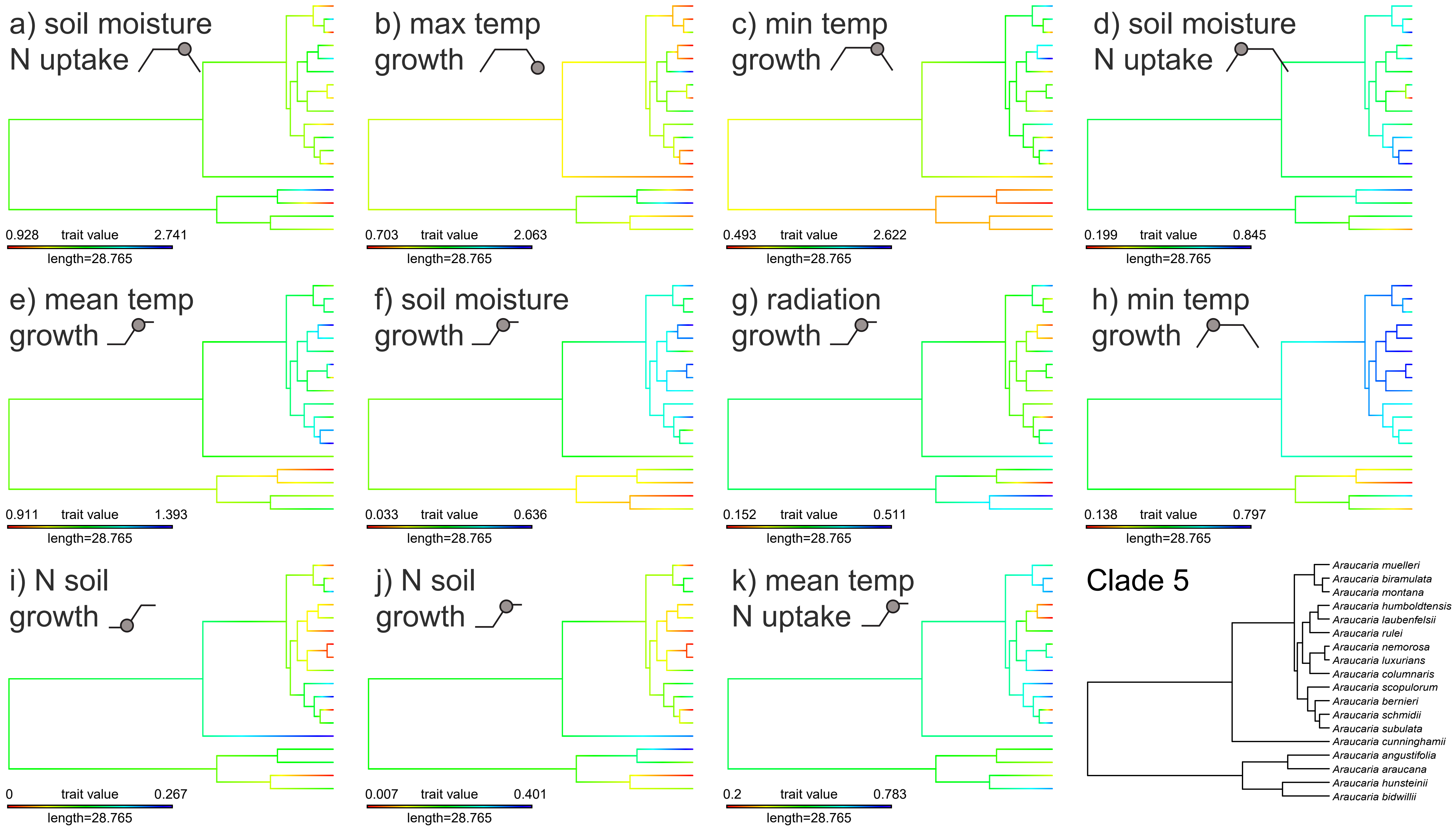

Supplementary Figure 3e

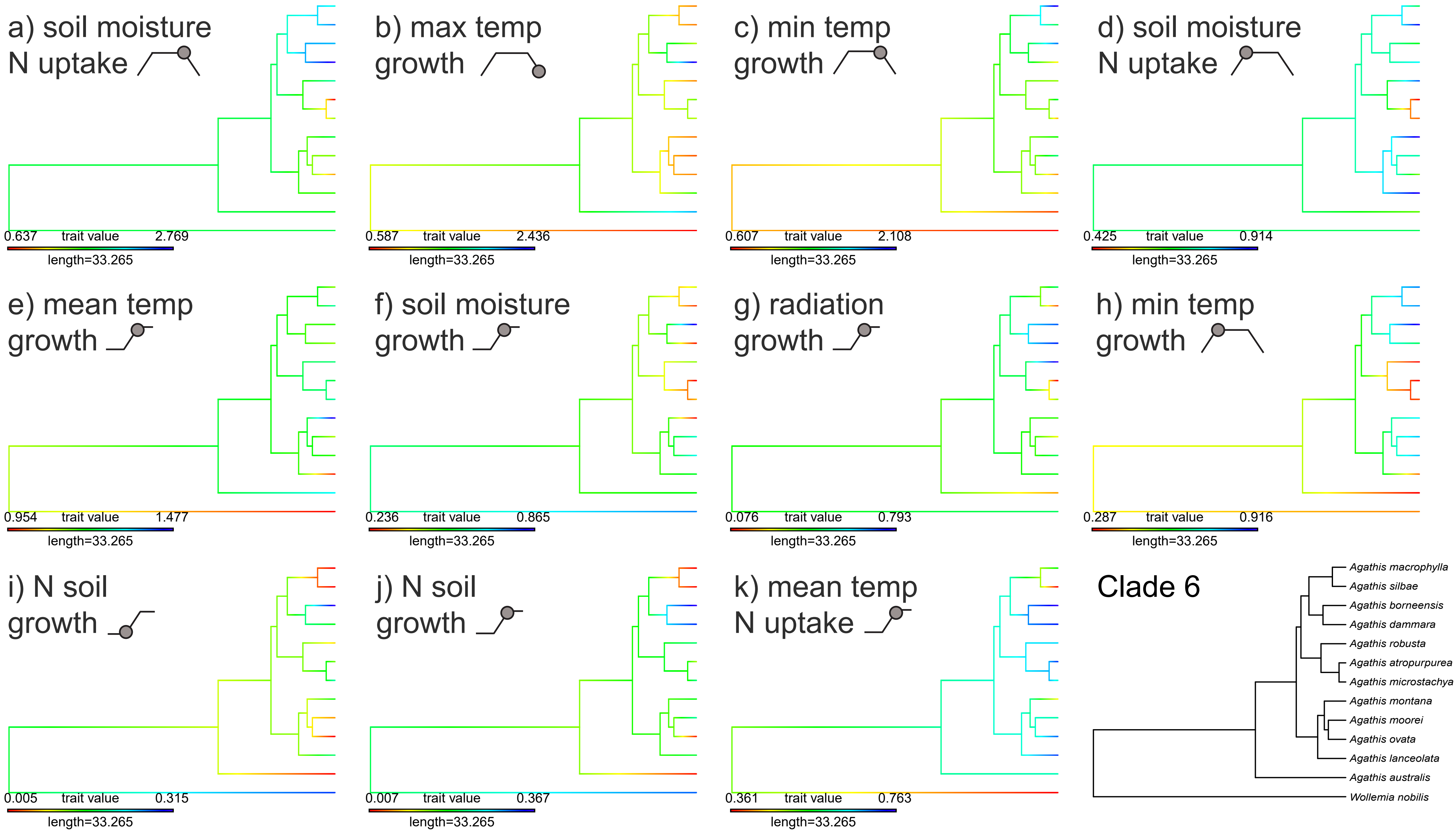

Supplementary Figure 3f

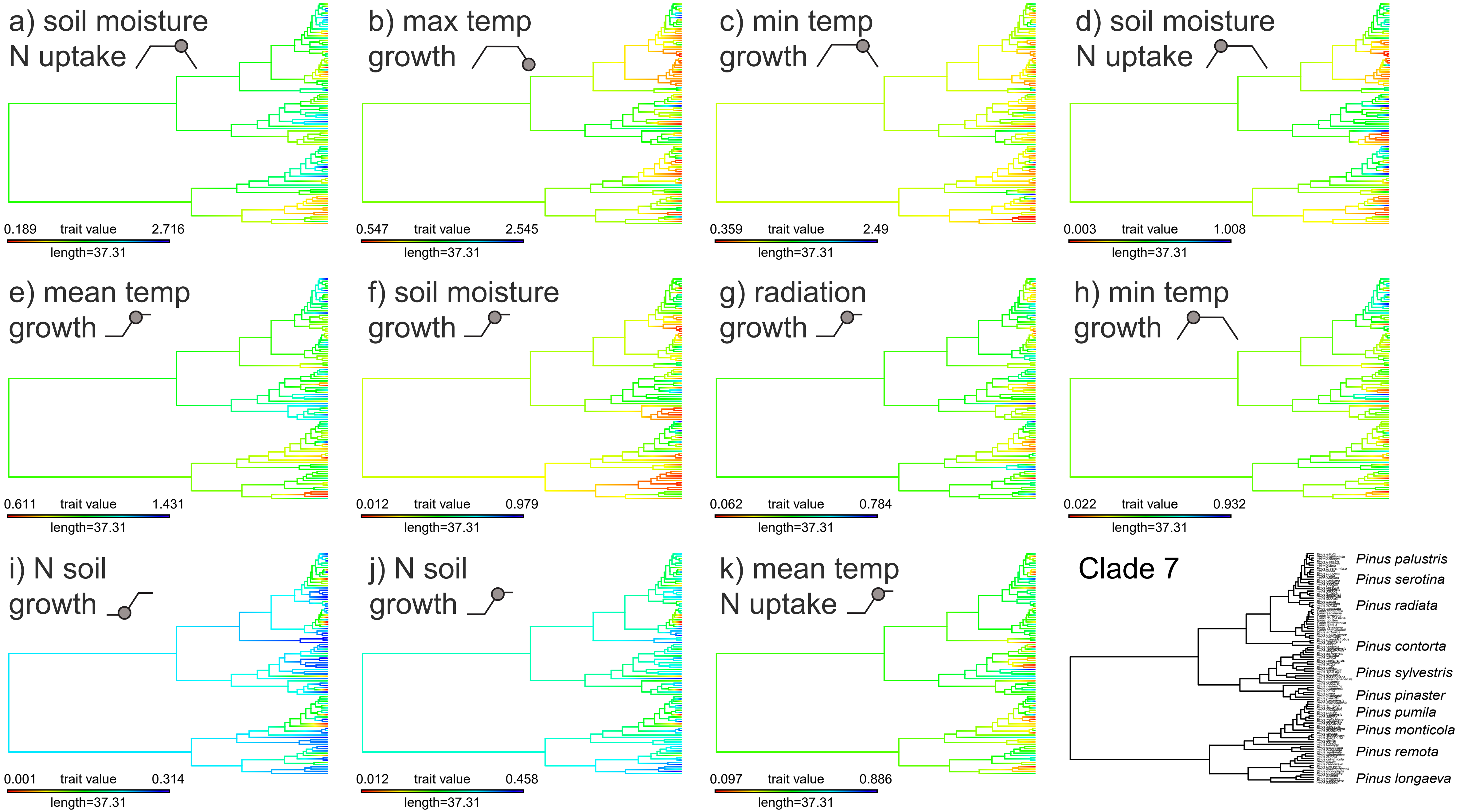

Supplementary Figure 3g

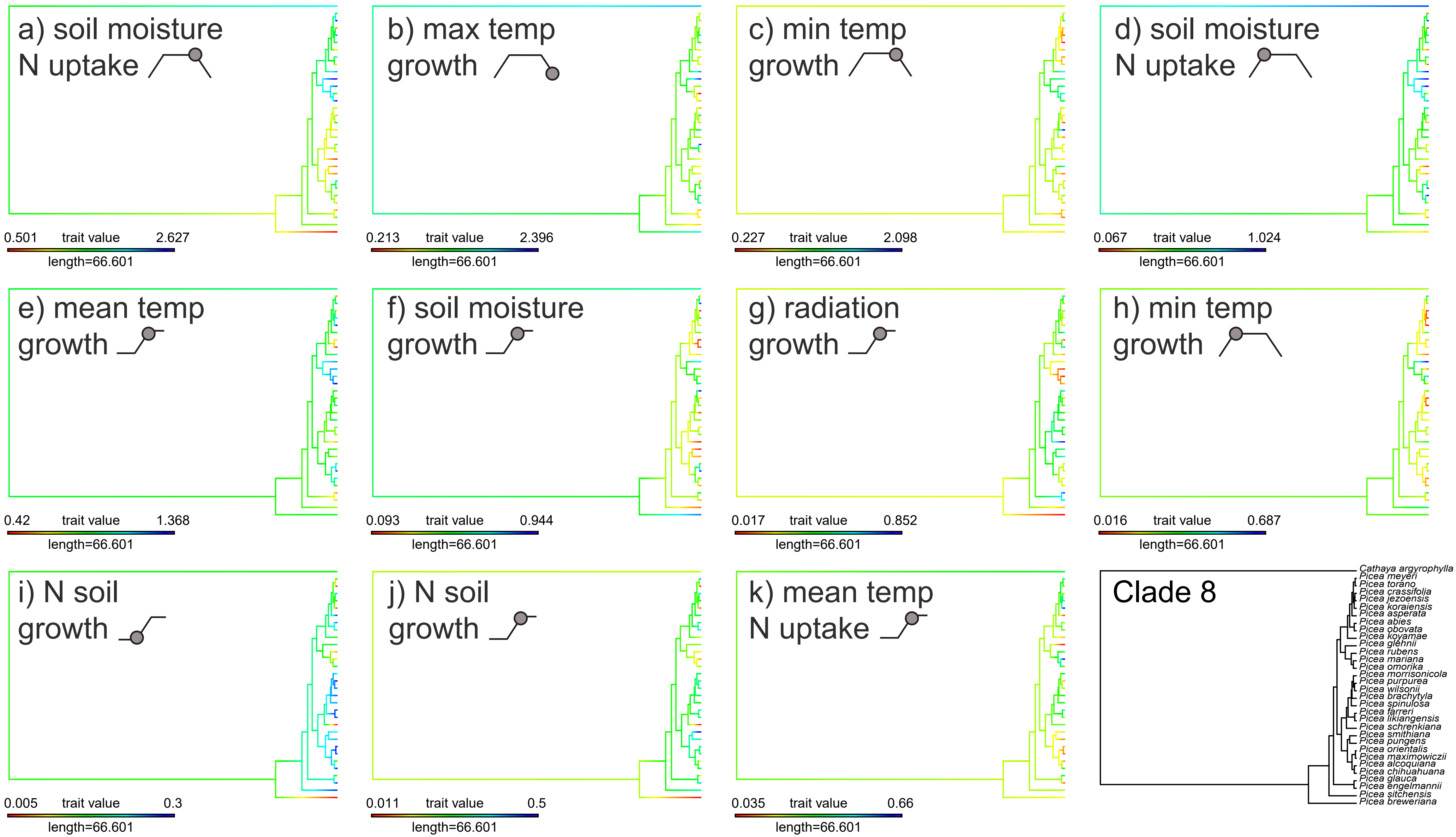

Supplementary Figure 3h

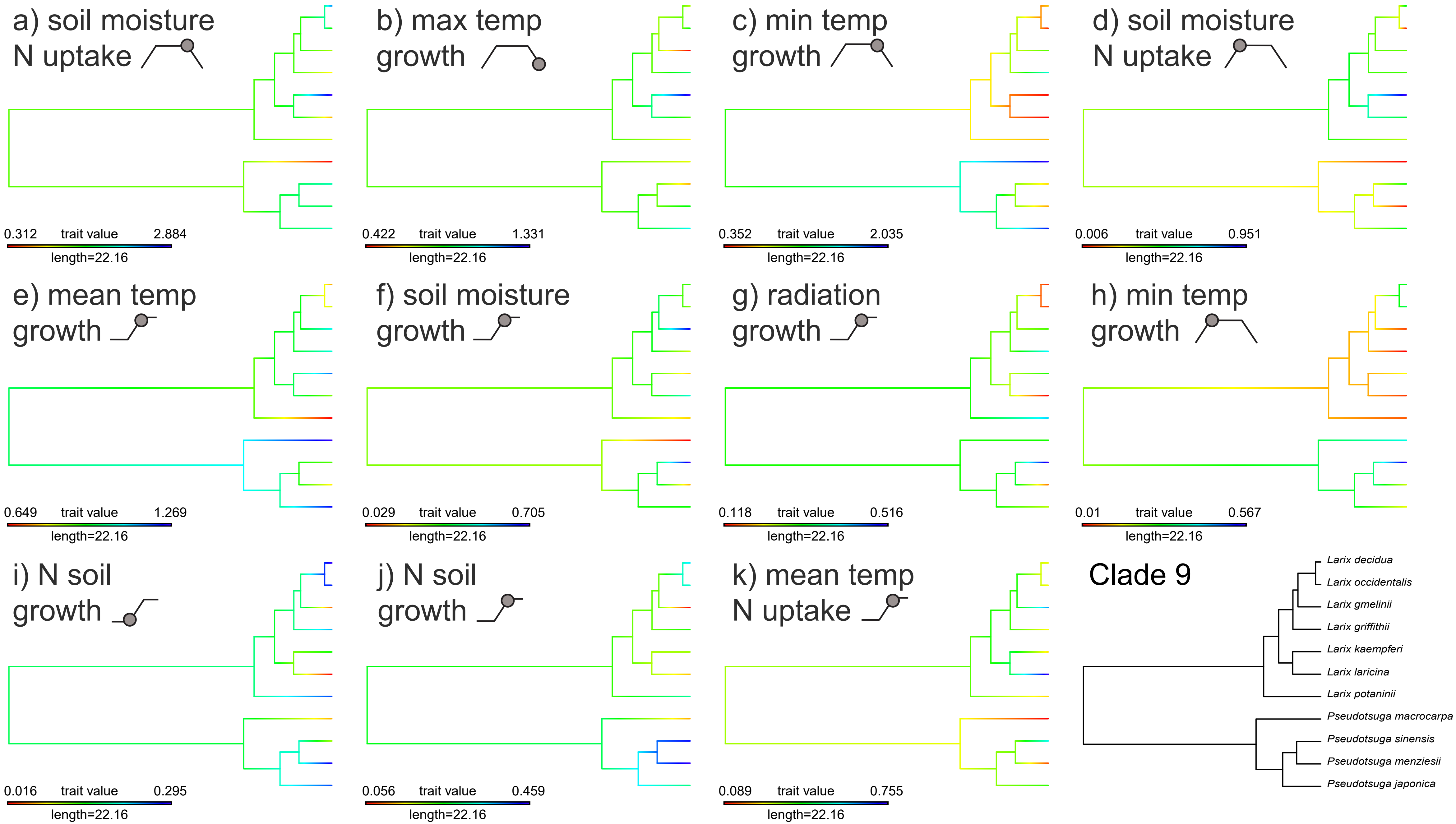

Supplementary Figure 3i

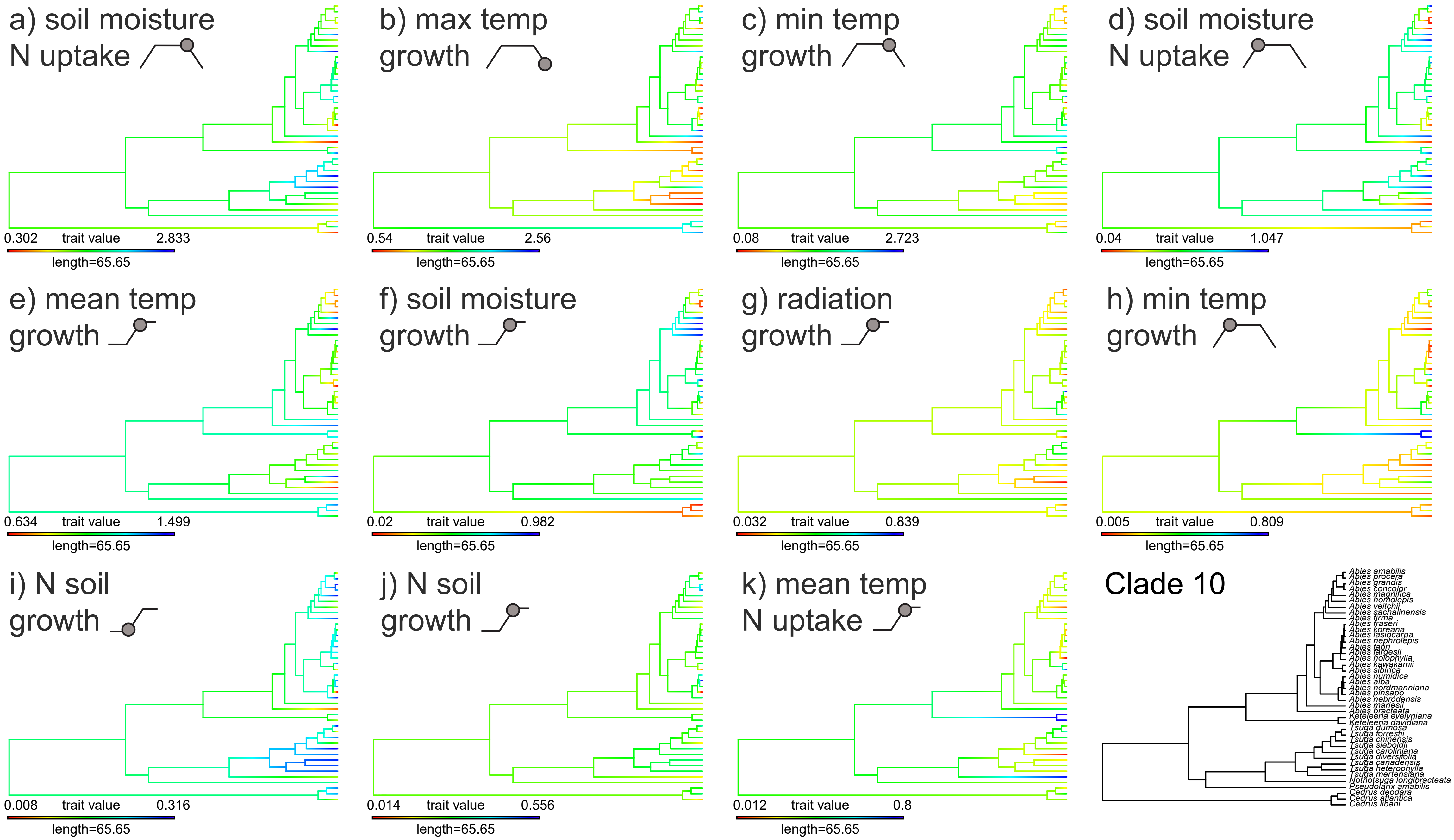

Supplementary Figure 3j

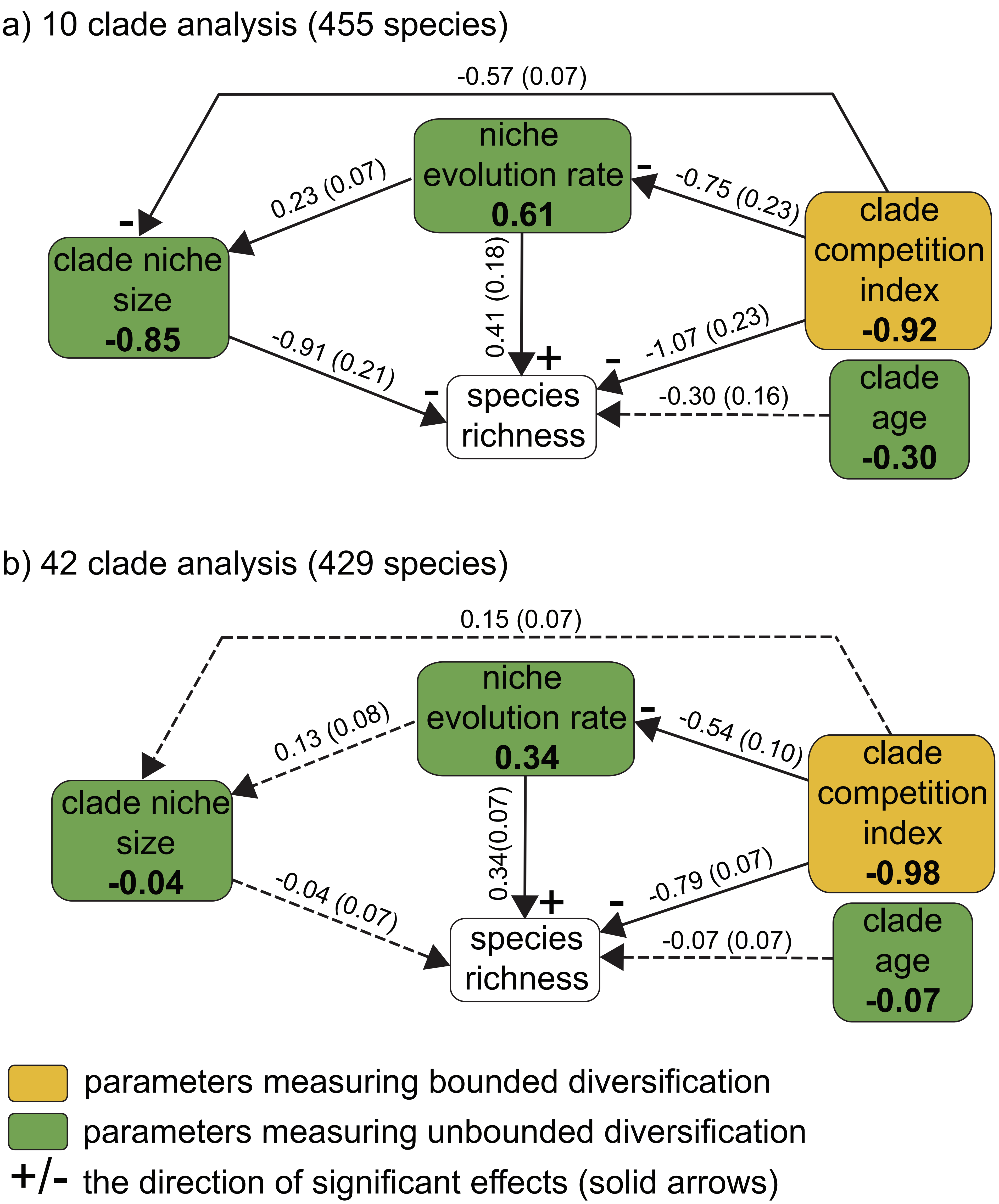

Supplementary Figure 4. Bayesian path analysis based on projecting the species niches into the real world, rather than a resampled version of the world where all environments are equally common as in Figure 3 (main text). Shown are the relative effects of niche and phylogenetic parameters on clade species richness for 455 conifer species in i) 10 large clades, and ii) 42 smaller clades. Total effect size is shown with the parameters, while direct effects and their standard deviation are shown along the vertices. Solid lines are significantly different from zero (95% credible intervals not including zero).

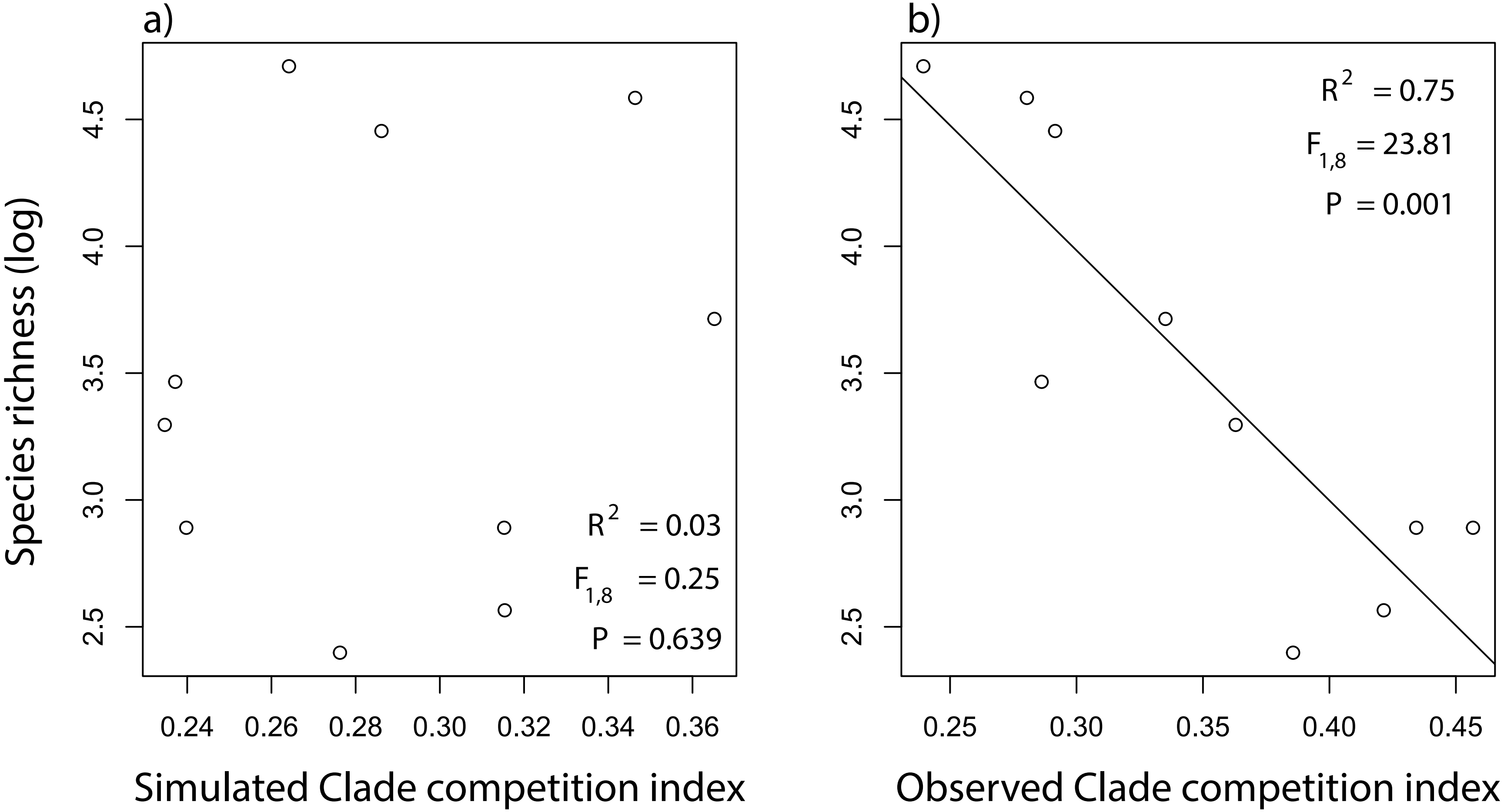

Supplementary Figure 5. Proof that the Clade competition index is unbiased in regard to species richness. a) The relationship between log-transformed species richness and simulated Clade competition index. Simulations involved calculating the clade competition index after randomly shuffling the species on the phylogeny (producing clades of unrelated species). The estimates presented are the mean of 10,000 simulations. b) The relationship between log species richness and the clade competition index calculated from the observed empirical data. If the Clade competition index is unbiased there should be no relationship between species richness and the simulated data. Least squares regression was used to test this prediction and the R^2, F-statistic (with degrees of freedom) and P-values are shown on the plots, clearly indicating that no relationship exists in a). In contrast, the solid line shows a significant linear relationship in b).

Supplementary Table 1. Differences in mean evolution rate of conifer niche traits at the 10 clade level compared using Phylogenetic Independent Contrast (PIC). The means were calculated from the 11 most important traits (see text) after they were converted to PICs and compared using ANOVA. Node contrasts correspond to the terminal nodes on the phylogeny in Fig. 5 (main text). Shown are the mean differences in PIC score, the Lower and Upper end point of the interval, and the P value after adjusting for multiple contrasts.

Node contrast	Difference in means	Lower	Upper	Adjusted P value
5-4	-1.341	-2.111	-0.571	<0.001
6-4	-1.919	-2.689	-1.148	<0.001
7-4	-0.667	-1.437	0.103	ns
8-4	-2.797	-3.568	-2.027	<0.001
9-4	-0.16	-0.93	0.61	ns
6-5	-0.577	-1.348	0.193	ns
7-5	0.674	-0.096	1.444	ns
8-5	-1.456	-2.226	-0.686	<0.001
9-5	1.182	0.411	1.952	<0.001
7-6	1.251	0.481	2.022	<0.001
8-6	-0.879	-1.649	-0.109	<0.05
9-6	1.759	0.989	2.529	<0.001
8-7	-2.13	-2.9	-1.36	<0.001
9-7	0.507	-0.263	1.278	ns
9-8	2.638	1.868	3.408	<0.001
