# Supplementary material for: The dimensionality of niche space allows bounded and unbounded processes to jointly influence diversification
Source: Nat Commun. 2018 Oct 15;9:4258. doi: 10.1038/s41467-018-06732-x (PMC6189034; doi:10.1038/s41467-018-06732-x)
Supplement: Supplementary file 2 — Description of Additional Supplementary Files [file 41467_2018_6732_MOESM2_ESM.pdf]

Description of Additional Supplementary Files

File Name: Supplementary Data 1
Description: Details of 455 conifer species used in the phylogenetic and physiological niche modelling to estimate drivers of diversification. Shown are: the clade calcification (10 and 42 clade); number of cleaned georeferenced presence records; the confusion matrix which describes the model fit in terms of true positives, true negatives, false positives and false negatives; and the estimated niche area in quarter degree grid squares for the globe (projected) and for version of the globe where all environmental zones are equally common (resampled), see Methods (main text) for further details.
